# Mind the gap! The mitochondrial control region and its power as a phylogenetic marker in echinoids

**DOI:** 10.1186/s12862-018-1198-x

**Published:** 2018-05-30

**Authors:** Omri Bronstein, Andreas Kroh, Elisabeth Haring

**Affiliations:** 1 Natural History Museum Vienna, Geological-Palaeontological Department, 1010 Vienna, Austria; 2 Natural History Museum Vienna, Central Research Laboratories, 1010 Vienna, Austria; 3 Department of Integrative Zoology, University of Vienna, Vienna, Austria

**Keywords:** Control region, Molecular phylogeny, Mitochondrial markers, NGS, Echinoidea, Sea urchins

## Abstract

**Background:**

In Metazoa, mitochondrial markers are the most commonly used targets for inferring species-level molecular phylogenies due to their extremely low rate of recombination, maternal inheritance, ease of use and fast substitution rate in comparison to nuclear DNA. The mitochondrial control region (CR) is the main non-coding area of the mitochondrial genome and contains the mitochondrial origin of replication and transcription.

While sequences of the *cytochrome oxidase subunit 1* (*COI*) and *16S rRNA* genes are the prime mitochondrial markers in phylogenetic studies, the highly variable CR is typically ignored and not targeted in such analyses. However, the higher substitution rate of the CR can be harnessed to infer the phylogeny of closely related species, and the use of a non-coding region alleviates biases resulting from both directional and purifying selection. Additionally, complete mitochondrial genome assemblies utilizing next generation sequencing (NGS) data often show exceptionally low coverage at specific regions, including the CR. This can only be resolved by targeted sequencing of this region.

**Results:**

Here we provide novel sequence data for the echinoid mitochondrial control region in over 40 species across the echinoid phylogenetic tree. We demonstrate the advantages of directly targeting the CR and adjacent tRNAs to facilitate complementing low coverage NGS data from complete mitochondrial genome assemblies. Finally, we test the performance of this region as a phylogenetic marker both in the lab and in phylogenetic analyses, and demonstrate its superior performance over the other available mitochondrial markers in echinoids.

**Conclusions:**

Our target region of the mitochondrial CR (1) facilitates the first thorough investigation of this region across a wide range of echinoid taxa, (2) provides a tool for complementing missing data in NGS experiments, and (3) identifies the CR as a powerful, novel marker for phylogenetic inference in echinoids due to its high variability, lack of selection, and high compatibility across the entire class, outperforming conventional mitochondrial markers.

**Electronic supplementary material:**

The online version of this article (10.1186/s12862-018-1198-x) contains supplementary material, which is available to authorized users.

## Background

Over the past decade, the emerging field of massively parallel sequencing (aka next generation sequencing or NGS) has seen dramatic advances in methods and decrease in costs. In many fields and applications NGS technologies are rapidly replacing the more traditional Sanger sequencing [1]. Nevertheless, despite the tremendous contribution of NGS technologies to fields such as metagenomics, forensics and clinical diagnostics, these advances are not without limitations. For one, the associated error rate of NGS platforms (~ 0.1-15%) is higher and the read length generally shorter (35-700 bp for short read approaches) [2] than those obtained by traditional Sanger sequencing [3]. Moreover, data accumulating from miscellaneous NGS studies show reduced sequence coverage in non-random regions of both nuclear and mitochondrial genomes [4, 5]. In particular, regulatory regions, such as CpG islands, promoter and 5’-UTR regions have been shown to be particularly prone to reduced coverage and poorer SNP-calling performance on NGS platforms [4]. Consequently, Sanger sequencing will most likely remain an essential component in DNA sequence acquisition in the foreseeable future.

Non-coding DNA sequences are segments of an organism’s DNA that do not encode protein sequences. While some non-coding DNA is transcribed into functional non-coding RNA molecules (e.g., transfer RNAs, ribosomal RNAs, small nuclear RNAs, micro RNAs), other may function in transcriptional and translational regulation of protein-coding sequences or serve as the origin of DNA replication (to name a few possibilities). The mitochondrial control region (CR) is the longest non-coding region in animal mitochondrial DNA (mtDNA), and is considered the most variable region of the mitochondrial genome [6]. Within the CR, the displacement loop (or D-loop), which is often synonymously used in the literature with CR [7], is in fact a region within the CR comprising a third strand of DNA creating a semi-stable structure [8]. It is this region of the CR that is considered most polymorphic.

Apart from the general advantages of mitochondrial markers in animal phylogenetic studies, namely their maternal inheritance, lack of recombination, and fast rate of evolution [9, 10], several unique qualities make the CR a favoured marker sequence for genetic diversity analyses, in particular, its exceptionally fast evolutionary rate (even in comparison to the rest of the mitochondrial genome [11, 12]), polymorphic nature [13] and presumed selective neutrality as a non-coding region (but see [14, 15]). Consequently, this region has been widely used as a genetic marker in phylogenetic studies of various animals including vertebrate classes such as fish (e.g., [16, 17]), amphibians [18], reptiles [19], birds [20] and mammals [21, 22] as well as numerous invertebrate taxa (e.g., [23–26]. Nevertheless, despite being extremely useful for some species, several factors may hinder the utility of this marker in others. One or several repeat regions within the CR have been found in some species and these may have detrimental effects on PCR amplification, sequencing, or both [27, 28]. Furthermore, some species exhibit segmental duplications involving the CR (e.g., [25, 29, 30]), that, in some cases, leads to the formation of pseudogenes that may be co-amplified by PCR [31, 32]. Further problems might arise from possible homogenization between duplicated copies of the CR (e.g., [32, 33]). Many researchers have, therefore, avoided using this region for phylogenetics, focusing instead on protein or ribosomal RNA coding genes [34–36], and only rarely has the inferential potential of the CR been evaluated in comparison to coding regions (e.g., [37, 38]).

In echinoderms, as in most other taxa, phylogenetic studies have mainly exploited a limited set of markers. The greater majority of studies utilised fragments of two mitochondrial regions: the *cytochrome c oxidase subunit 1* gene (*COI*) and the *16S ribosomal RNA* gene (*16S*) (e.g., [34–36, 39–45]). Indeed, despite the growing number of echinoderm molecular genetic studies over the past two decades, the limited variety of available markers, and in particular of markers applicable to a broad range of species (often referred to as ‘universal primers’), left many gaps in the echinoderm phylogenetic tree. This situation persists even when restricting the discussion to echinoids. Ward et al. [36] for example, utilising a fragment of the *COI* gene, encountered amplification problems for about 10% of their species. Jeffery et al. [34] failed to amplify certain species altogether and had pseudogene complications with others. Smith and Kroh [42] highlighted the incompleteness of available DNA sequence data in camarodonts in their analyses of two nuclear genes (*18S* and *28S rRNA* genes) and the two mitochondrial genes (*COI* and *16S*). Interestingly, the latter authors also stated that “*COI data in isolation found radically different branching orders within individual camarodont families, and placed some bona fide echinometrids as basal members of the Strongylocentridae clade*”, emphasizing the need for critical consideration of analyses solely hinging on *COI* data.

**Fig. 1:**
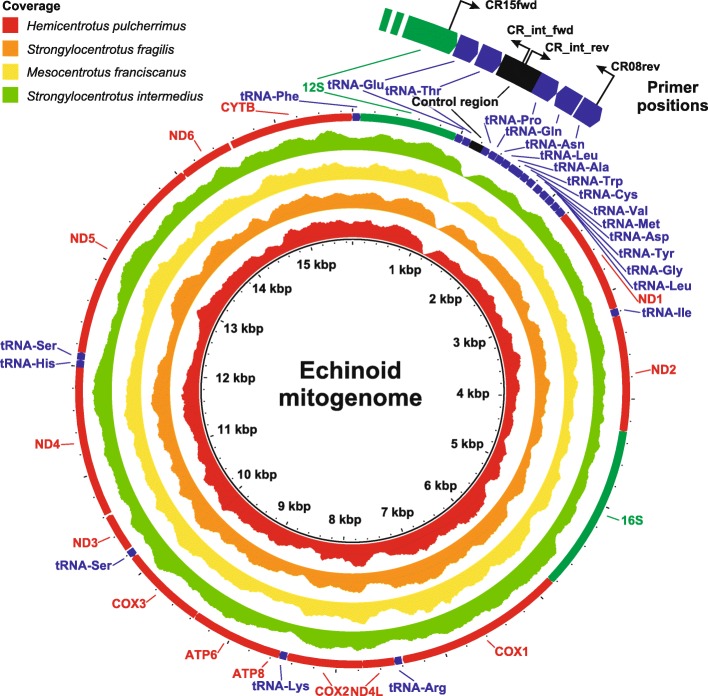
Representation of echinoid complete mitochondrial genomes assembled from NGS data, showing gene annotation and coverage. The annotated genomes are represented by four echinoid species: *Hemicentrotus pulcherrimus*, *Strongylocentrotus fragilis*, *Mesocentrotus franciscanus,* and *Strongylocentrotus intermedius*, corresponding to GenBank accession numbers: KC898202, KC898198, KC898199, and KC898200, respectively. Annotations are given at the outer margin of the external circle. Concentric circles represent the corresponding coverage for each of the represented species mitogenomes. Data was obtained from Kober and Bernardi [86, 87]. Enlarged segment illustrates the position of the various primers used in the current study. Coverage was calculated in BRIG [88], after read mapping with Bowtie2 [89] (using the predefined alignment threshold “very-sensitive”). Annotations are based on those for *H. pulcherrimus* (GenBank accession no. NC_023771) and radial plots generated using BRIG

Similar to other groups of organisms, NGS data in echinoids suffers from markedly reduced coverage in the CR area (Fig. 1), decreasing the quality and completeness of echinoid mitochondrial genome assemblies. Here we present the development and usability of a new set of primers targeting the echinoid CR and adjacent tRNAs (hereafter termed “CRA”, for CR and adjacent areas) to facilitate completing mitogenome assemblies based on NGS data, which often are characterized by low coverage in the control region. We demonstrate the high applicability of this region across the Echinoidea, both in the lab and in resulting phylogenies, and provide a phylogenetic analysis of a wide range of echinoid families based on this marker. Additionally, we utilise data extracted from all publicly available echinoid mitogenomes to evaluate the performance of the two most commonly used phylogenetic markers in echinoid studies (*COI* and *16S*) and compare them to both the CRA and the well-established echinoid morphological consensus phylogeny. CR sequence data hold great prospects for advancing our knowledge of echinoid phylogeny, of both distant and closely related species.

**Table 1:** Detailed taxonomic placement and GenBank accession numbers of taxa included in the current study

Order, Family	Genus	Species	*COI*	*16S*	CRA	Voucher No.
Cidaroida, Cidaridae	*Cidaroid*	sp.	–	–	MG198151^b,d^	MNHN IE-2007-3745
Cidaroida, Cidaridae	*Eucidaris*	*metularia*	–	–	MG198152^b,d^	SMNH Ec 25,624
Cidaroida, Cidaridae	*Prionocidaris*	sp.	–	–	MG198153^b,d^	MNHN IE-2007-3764
Cidaroida, Cidaridae	*Prionocidaris*	sp.	–	–	MG198154^b,d^	MNHN IE-2013-8705
Echinothurioida, Echinothuriidae	*Araeosoma*	*splendens*	–	–	MG198163^b,d^	MNHN IE-2007-1143
Echinothurioida, Echinothuriidae	*Asthenosoma*	*varium*	–	–	MG198164^b,d^	SMNH Ec 25,628
Diadematoida, Diadematidae	*Diadema*	*setosum*	KX385835^c,b^	KX385835^c,b^	KX385835^c,b^	
Diadematoida, Diadematidae	*Diadema*	*setosum*	–	–	MG198159^b,d^	SMNH Ec 25,437
Diadematoida, Diadematidae	*Diadema*	*setosum*	–	–	MG198160^b,d^	SMNH Ec 25,625
Diadematoida, Diadematidae	*Diadema*	*setosum*	–	–	MG198161^b,d^	SMNH Ec 25,626
Diadematoida, Diadematidae	*Diadema*	*setosum*	–	–	MG198162^b,d^	SMNH Ec 25,627
Diadematoida, Diadematidae	*Echinothrix*	*diadema*	KX385836^c^	KX385836^c^	KX385836^c^	
Micropygoida, Micropygidae	*Micropyga*	*tuberculata*	–	–	MG198165^b,d^	MNHN IE-2007-1152
Salenioida, Saleniidae	*Salenia*	*phoinissa*	–	–	MG198166^b,d^	MNHN IE-2007-3765
Stomopneustoida, Glyptocidaridae	*Glyptocidaris*	*crenularis*	KX638403^c,a^	KX638403^c,a^	KX638403^c,a^	
Arbacioida, Arbaciidae	*Arbacia*	*lixula*	X80396^c,b^	X80396^c,b^	X80396^c,b^	
Camarodonta, Echinidae	*Sterechinus*	*neumayeri*	KJ680295^c^	KJ680295^c^	KJ680295^c^	
Camarodonta, Echinidae	*Sterechinus*	*neumayeri*	KF214257^c,a^	KF214257^c,a^	KF214257^c,a^	
Camarodonta, Echinometridae	*Echinometra*	*mathaei*	KJ680291^c^	KJ680291^c^	KJ680291^c^	
Camarodonta, Echinometridae	*Echinometra*	sp.	–	–	MG198122^b,d^	SMNH OB ZNZ16
Camarodonta, Echinometridae	*Echinometra*	sp.	–	–	MG198123^b,d^	SMNH OB ZNZ37
Camarodonta, Echinometridae	*Echinometra*	sp.	–	–	MG198124^b,d^	SMNH OB ZNZ54
Camarodonta, Echinometridae	*Heliocidaris*	*crassispina*	KC479025^c^	KC479025^c^	KC479025^c^	
Camarodonta, Echinometridae	*Heterocentrotus*	*mammillatus*	KJ680292^c^	KJ680292^c^	KJ680292^c^	
Camarodonta, Echinometridae	*Zenocentrotus*	*kellersi*	–	–	MG198150^b,d^	USNM E40502
Camarodonta, Parechinidae	*Loxechinus*	*albus*	JX888466^c,b^	JX888466^c,b^	JX888466^c,b^	
Camarodonta, Parechinidae	*Loxechinus*	*albus*	KC490910^c,b^	KC490910^c,b^	KC490910^c,b^	
Camarodonta, Parechinidae	*Paracentrotus*	*lividus*	J04815^c,b^	J04815^c,b^	J04815^c,b^	
Camarodonta, Parechinidae	*Paracentrotus*	*lividus*	–	–	MG198127^b,d^	SMNH Ec 25,622
Camarodonta, Strongylocentrotidae	*Hemicentrotus*	*pulcherrimus*	KC898202^c,a^	KC898202^c,a^	KC898202^c,a^	
Camarodonta, Strongylocentrotidae	*Hemicentrotus*	*pulcherrimus*	KC490911^c,b^	KC490911^c,b^	KC490911^c,b^	
Camarodonta, Strongylocentrotidae	*Mesocentrotus*	*franciscanus*	KJ526170^c,a^	KJ526170^c,a^	KJ526170^c,a^	
Camarodonta, Strongylocentrotidae	*Mesocentrotus*	*nudus*	JX263663^c,b^	JX263663^c,b^	JX263663^c,b^	
Camarodonta, Strongylocentrotidae	*Mesocentrotus*	*nudus*	KC898201^c,a^	KC898201^c,a^	KC898201^c,a^	
Camarodonta, Strongylocentrotidae	*Pseudocentrotus*	*depressus*	KC490913^c^	KC490913^c^	KC490913^c^	
Camarodonta, Strongylocentrotidae	*Strongylocentrotus*	*droebachiensis*	EU054306^c,a^	EU054306^c,a^	EU054306^c,a^	
Camarodonta, Strongylocentrotidae	*Strongylocentrotus*	*droebachiensis*	AM900391^c,b^	AM900391^c,b^	AM900391^c,b^	
Camarodonta, Strongylocentrotidae	*Strongylocentrotus*	*fragilis*	KC898198^c,a^	KC898198^c,a^	KC898198^c,a^	
Camarodonta, Strongylocentrotidae	*Strongylocentrotus*	*intermedius*	KC490912^c^	KC490912^c^	KC490912^c^	
Camarodonta, Strongylocentrotidae	*Strongylocentrotus*	*intermedius*	KC898200^c,a^	KC898200^c,a^	KC898200^c,a^	
Camarodonta, Strongylocentrotidae	*Strongylocentrotus*	*pallidus*	AM900392^c^	AM900392^c^	AM900392^c^	
Camarodonta, Strongylocentrotidae	*Strongylocentrotus*	*purpuratus*	X12631^c,b^	X12631^c,b^	X12631^c,b^	
Camarodonta, Temnopleuridae	*Mespilia*	*globulus*	KJ680293^c^	KJ680293^c^	KJ680293^c^	
Camarodonta, Temnopleuridae	*Microcyphus*	*rousseaui*	–	–	MG198126^b,d^	SMNH Ec 25,621
Camarodonta, Temnopleuridae	*Salmacis*	*bicolor bicolor*	–	–	MG198130^b,d^	SMNH Ec 25,623
Camarodonta, Temnopleuridae	*Salmacis*	*bicolor rarispina*	KU302104^c^	KU302104^c^	KU302104^c^	
Camarodonta, Temnopleuridae	*Salmacis*	*sphaeroides*	KU302103^c^	KU302103^c^	KU302103^c^	
Camarodonta, Temnopleuridae	*Temnopleurus*	*hardwickii*	KP070768^c,b^	KP070768^c,b^	KP070768^c,b^	
Camarodonta, Temnopleuridae	*Temnopleurus*	*reevesii*	KU302106^c^	KU302106^c^	KU302106^c^	
Camarodonta, Temnopleuridae	*Temnopleurus*	*toreumaticus*	KU302105^c^	KU302105^c^	KU302105^c^	
Camarodonta, Toxopneustidae	*Lytechinus*	*variegatus*	–	–	MG198125^b,d^	SMNH Ec 25,620
Camarodonta, Toxopneustidae	*Pseudoboletia*	*indiana*	–	–	MG198128^b,d^	AIM MA 121531.1
Camarodonta, Toxopneustidae	*Pseudoboletia*	*indiana*	–	–	MG198129^b,d^	AIM MA 121531.2
Camarodonta, Toxopneustidae	*Tripneustes*	*depressus*	–	–	MG198131^b,d^	NHMW-DNAtis_26362
Camarodonta, Toxopneustidae	*Tripneustes*	*depressus*	–	–	MG198132^b,d^	NHMW-DNAtis_26363
Camarodonta, Toxopneustidae	*Tripneustes*	*depressus*	–	–	MG198133^b,d^	NHMW-DNAtis_26364
Camarodonta, Toxopneustidae	*Tripneustes*	*depressus*	–	–	MG198134^b,d^	NHMW-DNAtis_26365
Camarodonta, Toxopneustidae	*Tripneustes*	*depressus*	–	–	MG198135^b,d^	NHMW-DNAtis_26366
Camarodonta, Toxopneustidae	*Tripneustes*	*depressus*	–	–	MG198136^b,d^	NHMW-DNAtis_26367
Camarodonta, Toxopneustidae	*Tripneustes*	*gratilla gratilla*	KY268294^c,a^	KY268294^c,a^	KY268294^c,a^	
Camarodonta, Toxopneustidae	*Tripneustes*	*gratilla gratilla*	KJ680294^c^	KJ680294^c^	KJ680294^c^	
Camarodonta, Toxopneustidae	*Tripneustes*	*gratilla gratilla*	–	–	KY515261^b^	
Camarodonta, Toxopneustidae	*Tripneustes*	*gratilla gratilla*	–	–	KY515262^b^	
Camarodonta, Toxopneustidae	*Tripneustes*	*gratilla gratilla*	–	–	KY515263^b^	
Camarodonta, Toxopneustidae	*Tripneustes*	*gratilla gratilla*	–	–	KY515264^b^	
Camarodonta, Toxopneustidae	*Tripneustes*	*gratilla gratilla*	–	–	MG198149^b,d^	CAS 187197
Camarodonta, Toxopneustidae	*Tripneustes*	*gratilla gratilla*	–	–	MG198137^b,d^	NHMW 2017/0125/0001
Camarodonta, Toxopneustidae	*Tripneustes*	*gratilla gratilla*	–	–	MG198138^b,d^	NHMW 2017/0125/0003
Camarodonta, Toxopneustidae	*Tripneustes*	*gratilla gratilla*	–	–	MG198139^b,d^	NHMW 2017/0125/0004
Camarodonta, Toxopneustidae	*Tripneustes*	*gratilla gratilla*	–	–	MG198140^b,d^	NHMW Ev 20,497
Camarodonta, Toxopneustidae	*Tripneustes*	*gratilla gratilla*	–	–	MG198141^b,d^	NHMW Ev 20,498
Camarodonta, Toxopneustidae	*Tripneustes*	*gratilla gratilla*	–	–	MG198142^b,d^	NHMW Ev 20,499
Camarodonta, Toxopneustidae	*Tripneustes*	*gratilla gratilla*	–	–	MG198143^b,d^	NHMW 2016/0329/0001
Camarodonta, Toxopneustidae	*Tripneustes*	*gratilla gratilla*	–	–	MG198144^b,d^	NHMW 2016/0329/0002
Camarodonta, Toxopneustidae	*Tripneustes*	*gratilla gratilla*	–	–	MG198145^b,d^	NHMW 2016/0329/0003
Camarodonta, Toxopneustidae	*Tripneustes*	*gratilla gratilla*	–	–	MG198146^b,d^	NHMW 2016/0329/0004
Camarodonta, Toxopneustidae	*Tripneustes*	*gratilla gratilla*	–	–	MG198147^b,d^	NHMW Ev 20,500
Camarodonta, Toxopneustidae	*Tripneustes*	*gratilla gratilla*	–	–	MG198148^b,d^	NHMW Ev 20,501
Camarodonta, Toxopneustidae	*Tripneustes*	*gratilla elatensis*	–	–	KY515254^b^	
Camarodonta, Toxopneustidae	*Tripneustes*	*gratilla elatensis*	–	–	KY515255^b^	
Camarodonta, Toxopneustidae	*Tripneustes*	*gratilla elatensis*	–	–	KY515256^b^	
Camarodonta, Toxopneustidae	*Tripneustes*	*gratilla elatensis*	–	–	KY515257^b^	
Camarodonta, Toxopneustidae	*Tripneustes*	*gratilla elatensis*	–	–	KY515258^b^	
Camarodonta, Toxopneustidae	*Tripneustes*	*gratilla elatensis*	–	–	KY515259^b^	
Camarodonta, Toxopneustidae	*Tripneustes*	*gratilla elatensis*	–	–	KY515260^b^	
Camarodonta, Toxopneustidae	*Tripneustes*	*kermadecensis*	–	–	KY515241^b^	
Camarodonta, Toxopneustidae	*Tripneustes*	*kermadecensis*	–	–	KY515242^b^	
Camarodonta, Toxopneustidae	*Tripneustes*	*kermadecensis*	–	–	KY515243^b^	
Camarodonta, Toxopneustidae	*Tripneustes*	*kermadecensis*	–	–	KY515244^b^	
Camarodonta, Toxopneustidae	*Tripneustes*	*kermadecensis*	–	–	KY515245^b^	
Camarodonta, Toxopneustidae	*Tripneustes*	*kermadecensis*	–	–	KY515246^b^	
Camarodonta, Toxopneustidae	*Tripneustes*	*kermadecensis*	–	–	KY515247^b^	
Camarodonta, Toxopneustidae	*Tripneustes*	*kermadecensis*	–	–	KY515248^b^	
Camarodonta, Toxopneustidae	*Tripneustes*	*kermadecensis*	–	–	KY515249^b^	
Camarodonta, Toxopneustidae	*Tripneustes*	*kermadecensis*	–	–	KY515250^b^	
Camarodonta, Toxopneustidae	*Tripneustes*	*kermadecensis*	–	–	KY515251^b^	
Camarodonta, Toxopneustidae	*Tripneustes*	*kermadecensis*	–	–	KY515240^b^	
Camarodonta, Toxopneustidae	*Tripneustes*	*kermadecensis*	–	–	KY515252^b^	
Camarodonta, Toxopneustidae	*Tripneustes*	*kermadecensis*	–	–	KY515253^b^	
Clypeasteroida, Clypeasteroidae	*Arachnoides*	sp.	–	–	MG198155^b,d^	QM NO1_F4
Clypeasteroida, Clypeasteroidae	*Arachnoides*	sp.	–	–	MG198156^b,d^	QM NO1_F4B
Clypeasteroida, Clypeasteroidae	*Clypeaster*	*rarispinus*	–	–	MG198157^b,d^	MNHN IE-2013-8702
Clypeasteroida, Clypeasteroidae	*Clypeaster*	*rarispinus*	–	–	MG198158^b,d^	MNHN IE-2013-8704
Spatangoida, Loveniidae	*Echinocardium*	*cordatum*	FN562581^c,b^	FN562581^c,b^	FN562581^c,b^	
Spatangoida, Maretiidae	*Nacospatangus*	*alta*	KC990834^c,b^	KC990834^c,b^	KC990834^c,b^	
Spatangoida, Pericosmidae	*Pericosmus*	*bidens*	–	–	MG198167^b,d^	MNHN IE-2007-1138
Spatangoida, Pericosmidae	*Pericosmus*	*bidens*	–	–	MG198168^b,d^	MNHN IE-2013-8706
Spatangoida, Pericosmidae	*Pericosmus*	*bidens*	–	–	MG198169^b,d^	MNHN IE-2013-8707
Spatangoida, Pericosmidae	*Pericosmus*	*bidens*	–	–	MG198170^b,d^	MNHN IE-2013-8708

*COI - cytochrome* c oxidase subunit I, *16S rRNA –* ribosomal RNA, *CRA* -control region area (including the control, adjacent *tRNA*s and a part of the *12S rRNA* genes). Sequence type indicates whether the source sequence was generated by Sanger or next generation sequencing

^a^sequence data generated by NGS

^b^sequence data generated by Sanger sequencing

^c^data retrieved from complete mitochondrial genome sequence

^d^sequences generated in the current study

*AIM* Auckland War Memorial Museum, *CAS* California Academy of Sciences, *MNHN* Muséum national d'Histoire naturelle, *NHMW* Natural History Museum Vienna, *QM* Queensland Museum, *SMNH* Steinhardt Museum of Natural History, *USNM* Smithsonian Institution National Museum of Natural History

## Methods

### Taxon sampling

Sequence data from 110 specimens were included in the current analysis. Forty-nine of these sequences were generated as part of the current study, and the remaining were obtained from GenBank (Table 1). Newly generated sequences were based on specimens deposited in museum collections as listed in Table 1 and comply with institutional, national, and international guidelines. The sequences obtained from GenBank included all 35 currently available complete echinoid mitochondrial genomes comprising both NGS and Sanger generated sequences. To evaluate the performance of our target region as a phylogenetic marker and the applicability of the primers across the Echinoidea, a broad range of echinoid taxa were sampled. Additionally, to test the applicability of the CRA primers for specimens at varying grades of preservation, we included material of varying quality, from freshly sampled tissue, through ethanol fixed collection material, to dried specimens nearly a century old. In total, 10 orders comprising 17 families, 34 genera and 45 species were represented in the current analysis (Table 1).

### Development of the CRA primers

Primers were designed to flank the control region and D-loop in order to retrieve the full length of this target region. To search for highly conserved regions adjacent to the CR, all publicly available echinoid complete mitochondrial genomes were downloaded, primarily aligned using MAFFT v. 7.2 [46] and subsequently adjusted by eye using Bioedit v. 7.1.3 [47] and AliView v. 1.18.1 [48]. The highly variable nature of the CR, prevented developing a set of ‘universal echinoid’ primers suitable for a broad range of echinoid species. Consequently, our final CRA target included the genes for *12S rRNA* (partially), *tRNA*^*Glu*^, *tRNA*^*Thr*^, the CR, *tRNA*^*Pro*^, *tRNA*^*Gln*^, and a partial sequence of *tRNA*^*Asn*^. Two sets of primers were developed: CR15fwd 5’-TACACATCGCCCGTCACTCT-3′ (positioned at the 3′ end of the *12S* gene) and CR08rev 5’-TTAACGGCCAAGCGCCTTT-3′ (binding within the *tRNA*^*Asn*^ gene), complemented by two internal primers: CR_int_fwd 5’-CTTTGGGAGTTGCAAATGTAAGTG-3′ and CR_int_rev 5’-TTTAACCCTCTCTCCTGGTTTACA-3′ (Fig. 1).

### Laboratory procedures

Total genomic DNA was extracted from tube feet and spine muscles using the DNeasy® Blood and Tissue Kit (QIAGEN) following the manufacturer’s instructions. PCR amplifications with the TopTaq DNA Polymerase (QIAGEN) were conducted using 1 μl of extracted genomic DNA (approximately 10-15 ng). Reaction conditions using primers CR15fwd and CR08rev were 3 min at 94 °C, followed by 35 cycles of 30 s at 94 °C, 30 s at 57 °C and 60 s at 72 °C, ending with a final extension step of 10 min at 72 °C. PCR products were visualized on a 1.5% agarose gel, purified using ExoSAP-IT (Affymetrix) and sent for sequencing to Microsynth GmbH (Vienna, Austria) using the PCR primers. In cases of weak amplifications (amplicons of the expected size showing only as faint bands on an agarose gel), target selected re-amplifications were performed following the methods of Bjourson and Cooper [49] using the same primers and reaction conditions as above. For this purpose, the respective PCR fragments were visualised on an agarose gel under UV light and templates for the re-amplification were transferred from the gel to the new PCR tubes using a sterile needle.

Despite yielding a product at the expected length, all amplicons failed to sequence through using the external PCR primers, with the sequencing signal dropping ca. half way through the expected amplicon length (see results for details). Consequently, the internal primers CR_int_fwd and CR_int_rev were used to generate a two-way sequencing of each amplicon (before and after the break-off). The sequences determined in the present study were deposited in GenBank under the accession numbers: MG198122–MG198170.

### Data assembly and phylogenetic analyses

Four datasets were created to facilitate our analyses. (1) *CRA-all* comprising all publicly available echinoid sequences in addition to the 49 sequences generated in the current study (405 bp long). To facilitate the direct comparisons of the CR performance as a phylogenetic marker with the two most common mitochondrial markers (*COI* and *16S*), three additional datasets were constructed based solely on the 35 publicly available complete echinoid mitogenomes: (2) *Mito-COI* was extracted from the mitogenomes targeting the region defined by the primers EchinoF1 and HCO2198 as in Ward et al. [36] yielding an alignment of 33 unique sequences, 562 bp long. (3) *Mito-16S* was extracted from the mitogenomes targeting the region defined by the primers 16sar-L and 16sbr-H of Palumbi (in [50]) yielding an alignment of 33 unique sequences, 558 bp long. (4) *Mito-CRA* was extracted from the mitogenomes targeting our current CR primers (CR15fwd and CR08rev), yielding an alignment of 33 unique sequences, 405 bp long. In all of the above datasets, ambiguous site removal was performed with trimAl v. 1.2 ([51]; setting: -*automated1)*, followed by unique sequences detection using DAMBE6 [52]. The phylogenies based on the above datasets were compared to the current working classification of echinoids as presented by Kroh and Smith [53] and implemented in the World Echinoidea Database [54]. This classification is based on a large-scale numerical cladistic analysis involving representatives of all extant and fossil echinoid families and more than 300 morphological characters.

Phylogenetic analyses were conducted using both Maximum Likelihood (ML) and Bayesian Inference (BI) approaches. A heuristic search under the Bayesian Information Criterion (BIC) [55], as implemented in PartitionFinder2 [56], was employed to determine the optimal partitioning schemes and models of molecular evolution for the phylogenetic analyses (Table 2).

**Table 2:** Sequences summary for the different datasets including best-fitting nucleotide substitution models

	*CRA-all*	*Mito-COI*	*Mito-16S*	*Mito-CRA*
Number of unique sequences	86	33	33	33
MSA length (bp)	405	562	558	405
%G	20.4	18.9	22.3	20.9
%A	30.9	26.0	31.0	30.7
%C	21.4	24.4	20.5	21.0
%T	27.3	30.8	26.2	27.5
P_inv_	0.09384	0.34741 (codon position 1&2) 0.04714 (codon position 3)	0.36998	0.17559
Overall mean K2P/p-distance	0.16/0.14	0.21/0.18	0.16/0.143	0.16/0.14
Best-fit model – (ML)	GTR + G	GTR + I + G	GTR + G	GTR + G
Best-fit model – (BI)	HKY + G	SYM + I + G	GTR + G	HKY + G

*ML* Maximum Likelihood, *BI* Bayesian Inference, *CR* Control region, *COI* cytochrome c oxidase subunit 1, *16S* 16S ribosomal RNA, *MSA* multiple sequence alignment, *P*_*inv*_ proportion of invariant sites

ML analysis was performed using the program RAxML GUI v. 1.5b1 [57]. Settings were ‘ML + thorough bootstrap’, 100 runs, 1000 replicates, applying the best-fit models as inferred from PartitionFinder2. Bayesian analysis was carried out using the program MrBayes v. 3.2.2 [58]. We ran two independent runs of three ‘heated’ and one ‘cold’ chain for 10 million generations and sampled parameters and trees every 100 generations. The runs were also inspected with Tracer v. 1.6 [59] to assess the behaviour of the runs and convergence was assessed using RWTY package [60] implemented in R v. 3.2.1 [61]. In a conservative approach, the first 25% of trees were discarded as burn-in and a 50% majority rule consensus tree was calculated from the remaining trees. Bayesian Posterior Probabilities (PP) were obtained from the 50% majority-rule consensus of the trees sampled during the stationary phase.

The topologies of the different phylogenetic trees were visualised and compared using Phylo.io [62], applying a variant of the Jaccard Index (Jaccard similarity coefficient) as the comparison metric as implemented in Phylo.io. This tool performs automated branch-swapping in order to find the best corresponding visualization between two trees and compares branching order. Additionally, trees were also compared using the duplication-aware algorithm treeKO [63] implemented in the ete-compare module of the Python Environment for Tree Exploration (ETE) [64]. In contrast to other algorithms for tree comparison, treeKO does not require complete and exact matching of terminal taxa between compared trees and provides Robinson-Foulds-based distance metrics even in the presence of duplication and loss events.

Substitution saturation decreases the amount of phylogenetic signal to the point that sequence similarities could as likely be the result of chance alone rather than homology. Consequently, when saturation is reached, phylogenetic signal is lost and the sequences are no longer informative about the underlying evolutionary process that produced them [65]. Saturation of substitutions was evaluated by plotting the number of transitions (s) and transversions (v) against the F84 [66] genetic distance, as well as by comparing the information entropy-based index (I_ss_) with critical values (I_ss.c_) [67, 68] as implemented in DAMBE6 [52]. If I_ss_ is significantly lower than I_ss.c_, sequences have not experienced substitution saturation.

## Results

### Primer performance and sequence characteristics

Amplifications of the CR and adjacent tRNAs performed well across the diverse groups of echinoids, generally yielding a single distinct product of the expected length (Additional file 1: Figure S1). Notably, arrangement of the mitochondrial genes around the CR (including the *12S* and various tRNA genes) as well as the features within the CR were identical in all of the taxa analysed as well as the taxa obtained from GenBank indicating a conserved organisation of this area. PCR amplifications performed equally well on DNA extracted from tissue of varying quality (considering tissue age and methods of preservation as outlined above). The oldest sample analysed in the current study was a dried specimen of *Zenocentrotus kellersi* A.H. Clark, 1931 (USNM E40502), collected in 1930 together with the holotype. DNA from this specimen was successfully amplified and sequenced, allowing for the determination of the phylogenetic position of *Z. kellersi*.

Regardless of successful amplifications, complete sequencing of amplicons in full length using only the external PCR primers failed. Sequencing using both the forward and reverse primers suffered an abrupt signal loss at a stretch of at least 12 guanine bases, roughly in the middle of the amplified region (see Additional file 1: Figure S2 and text below). Applying the internal primers enabled sequencing the complementary strand (before and after the guanine stretch) thereby ensuring a reliable two-way read of the amplified sections.

The echinoid CR is located within a cluster of 15 tRNA genes, between the genes for *tRNA*^*Th*r^ and *tRNA*^*Pro*^ confirming the gene order noted previously by Jacobs et al. [69]. This position was confirmed for all taxa examined in the current study. The middle of the CR is composed of a stretch of up to 20 guanine residues (referred to as the poly-G stretch) although the precise length of this string could not be unambiguously determined (due to the sequencing limitations discussed above). Nevertheless, some taxa consistently showed a shorter poly-G stretch than others. The shortest one was recovered in *Asthenosoma varium* and *Araeosoma splendens* (with 12 G residues) followed closely by the diadematoids *Diadema setosum* and *Echinothrix diadema*, both taxa consistently yielding 13 G residues. The 3′ side of the G stretch is followed by an A + T- rich segment that resembles the TATA box found in the promotor regions of eukaryotes. On the 5′ side of the G stretch is a heterogeneous yet fairly conserved segment of 37 to 40 bp in most cases, preceded by a polypyrimidine tract as observed by Jacobs et al. [69]. As before, the exceptions being the diadematoids which display a shorter, 20 bp segment, and echinothuroids which possess a 30 bp long heterogeneous segment prior to the polypyrimidine tract.

### Mitochondrial markers comparison

**Fig. 2:**
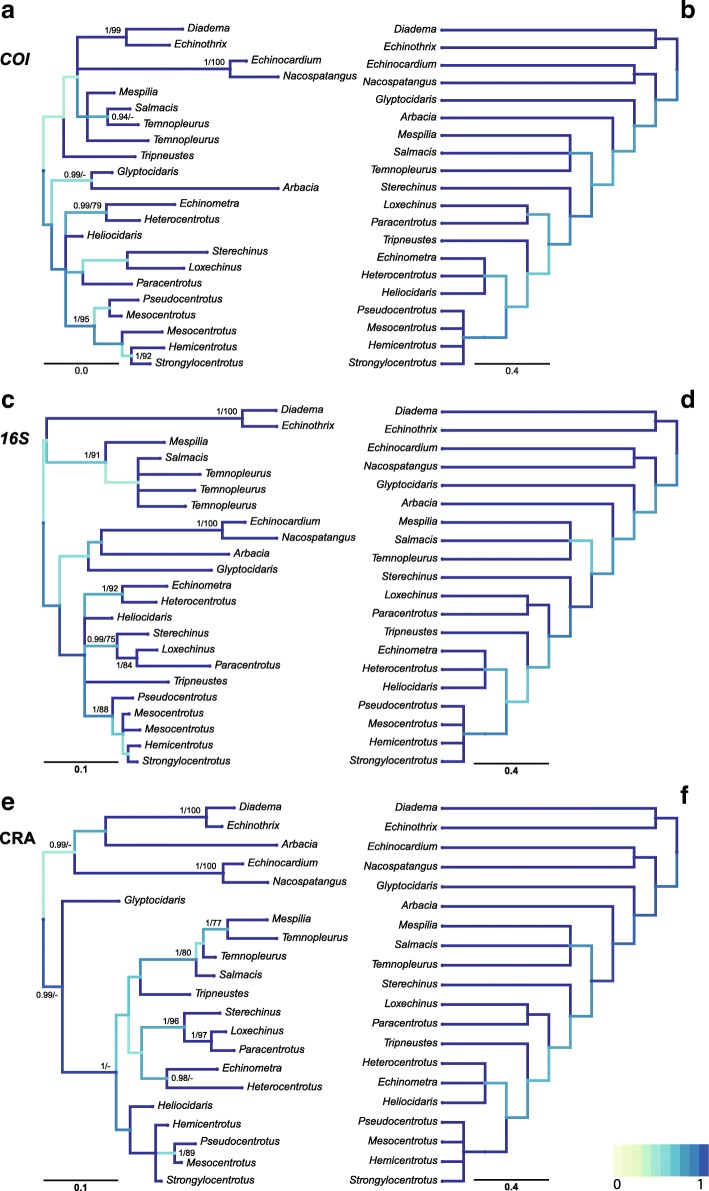
Pairwise tree comparisons for phylogenetic trees based on commonly used mitochondrial markers. Trees include the two most commonly used phylogenetic mitochondrial markers: a fragment of the *cytochrome c oxidase subunit 1* (**a**) gene and a fragment of the *16S ribosomal RNA *(**c**) as well as the novel tRNAs and control region (**e**). To facilitate independent comparisons, the genetically inferred trees were restricted to the 35 publicly available complete echinoid mitochondrial genomes. Genera represented by more than one species were collapsed and are depicted by single branches. Supporting values (> 0.85 posterior probabilities and > 75% ML bootstrap values) are shown next to nodes. Topological comparisons between the genetically inferred trees and current classification (**b**, **d**, **f**) (see text for details) were visualised using Phylo.io [62]. Colour scale for the comparison metric (a variant of the Jaccard Index as implemented in Phylo.io) ranges from 0 (subtrees completely different) to 1 (subtree structure of the respective node is identical)

Phylogenetic reconstructions of the three selected mitochondrial markers (i.e., *COI*, *16S* and CRA), extracted from the 35 complete echinoid mitochondrial genomes are shown in Fig. 2. Taxa from the above datasets were collapsed to genera. The corresponding tree for each marker (left column trees, denoted: A – *COI*, C – *16S* and E – CRA) is shown in comparison to the current [53, 54] echinoid working classification (right column trees, denoted: B, D and F). Topological similarities between the gene trees and the systematic consensus tree are highlighted using the variant Jaccard Index metric as implemented in Phylo.io [62]. The colour coded comparison metric with a score of 1 on the COI gene tree for example (Fig. 2a), denotes an identical subtree structure in the corresponding tree based on morphology (Fig. 2b).

All three markers show high congruence for some of the taxa but marked differences for others. At large, the CRA tree seem to be superior and outperform the other two markers in terms of deep divergence and accuracy in comparison to the systematic consensus tree. The *COI* tree for example misplaces the temnopleurids as the sister group of diadematoids and the Irregularia and places the latter two clades as the sister group of the camarodont *Tripneustes* (Fig. 2a) although these topologies are poorly supported. In the *16S* tree, the Irregularia are misplaced and included within the echinaceans while the temnopleurids are excluded from the latter and resolved as sister group of the diadematoids (Fig. 2c) yet once again this was poorly supported. In general, both *COI* and *16S* reconstructed topologies were inferior to the CRA in terms of resolution and branch support (Fig. 2). While the former topologies retrieved polytomies at the basal nodes of the Camarodonts (also observed in the analysis of Smith et al. [70]) as well as the temnopleurids, both were well resolved in the CRA tree (Fig. 2e). Nevertheless, some discrepancies also occurred within the CRA inferred topology, namely the misplacement of *Arbacia* (a sequence deriving from GenBank, not reconfirmed in the present study) as sister group of the diadematoids (with low support 0.8/−) with both being the sister group of the Irregularia. Interestingly, *Heliocidaris* was consistently misplaced outside the Echinometridae in all three data sets, either placed in a basal polytomy amongst members of the Camarodonta (*COI* and *16S*) or as sister group of the Strongylocentrotidae (CRA).

Summary statistics for the comparison of the different mitochondrial markers are given in Table 3. As the trees used in the current comparison were not strictly symmetric and contained duplicate attributes (i.e., tip names), only the duplication aware distance of the TreeKO method (treekoD) was appropriate for the comparison. Duplicated attributes were formed during the phylogenetic analysis as genera were recovered as non-monophyletic and split into several clades. The CRA tree had a significantly larger effective size in comparison to *COI* and *16S* (i.e., more items from this tree were used for the comparison with the systematic consensus tree). The treekoD metric showed similar values for all three markers, with the values for *COI* and CRA being virtually identical (0.60 and 0.61, respectively).

**Table 3:** Phylogenetic tree comparisons using the duplication-aware algorithm TreeKO as implemented in the Python Environment for Tree Exploration (ETE)

Source tree	Reference tree	Effective tree size	nRF	RF	maxRF	%src_br	%ref_br	Subtrees number	treekoD
*COI*	Syst	10.0	0.60	9	15	0.38	0.43	1	0.60
*16S*	Syst	11.0	0.53	9	17	0.44	0.50	4	0.53
CRA	Syst	18.5	0.61	18.5	31	0.36	0.43	2	0.61

Genetically inferred trees based on: *COI*, *16S*, CRA are compared to current echinoid classification (Syst) (see text for details)

*RF* Robinson-Foulds symmetric distance, *nRF* normalized RF distance (RF/maxRF); %src_br – frequency of edges in target tree found in the reference; %ref_br – frequency of edges in the reference tree found in the target; Subtrees – number of subtrees used for the comparison; treekoD – average p distance among all possible subtrees in the original target trees to the reference tree (lower treekoD values are indicative of higher similarity between trees)

**Table 4:** Substitution saturation analysis of the CRA region based on the index of substitution saturation as implemented in DAMBE6

Marker dataset	Number of OTU^a^	Iss^b^	I_ss.cSym_^c^	df^d^	*p* value^e^	I_ss.cAsym_^f^	df^d^	*p* value^e^
*CRA-All*	4	0.240	0.789	366	< 0.0001	0.757	366	< 0.0001
	8	0.259	0.743	366	< 0.0001	0.631	366	< 0.0001
	16	0.242	0.703	366	< 0.0001	0.494	366	< 0.0001
	32	0.260	0.692	366	< 0.0001	0.363	366	0.0001

^a^Number of sequences used in the random resampling

^b^index of substitution saturation

^c^critical value for a symmetrical tree topology

^d^degrees of freedom

^e^probability that I_ss_ is significantly different from the critical value (I_ss.cSym_/I_ss.cAsym_)

^f^critical value for an asymmetrical tree topology

Note: two-tailed tests are used

**Fig. 3:**
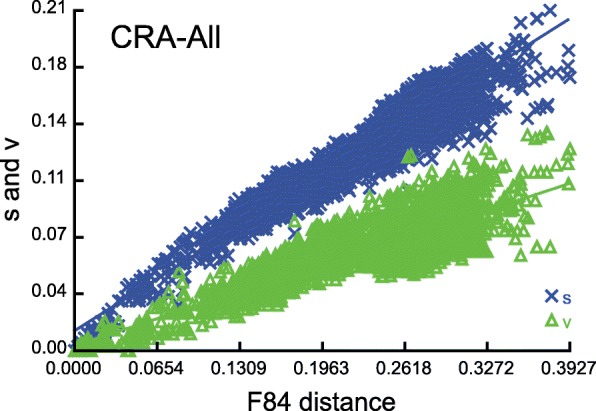
Substitution saturation plot of the CRA marker based on the *CRA-All* dataset. The number of transitions (s) and transversions (v) is plotted against F84 genetic distance. A linear correlation is sustained for both transitions and transversions as expected in the absence of saturation

Substitution saturation was evaluated for all markers and datasets. No saturation was detected in the CRA as reflected from the linear correlation of the number of transitions and transversions plotted against genetic distance (Fig. 3), as well as from a significantly lower value of I_ss_ in comparison to I_ss.c_ (for both symmetrical and asymmetrical weighed topologies) (Table 4). Concerning the *16S* marker, no saturation was detected assuming a symmetrical topology, while incipient saturation was detected assuming an asymmetrical topology for 32 OTUs and above (Additional file 1: Figure S3 and Table S1). In *COI* saturation were more prominent at the third codon position (Additional file 1: Figure S3 and Table S1).

### Echinoid phylogeny based on the CR

**Fig. 4:**
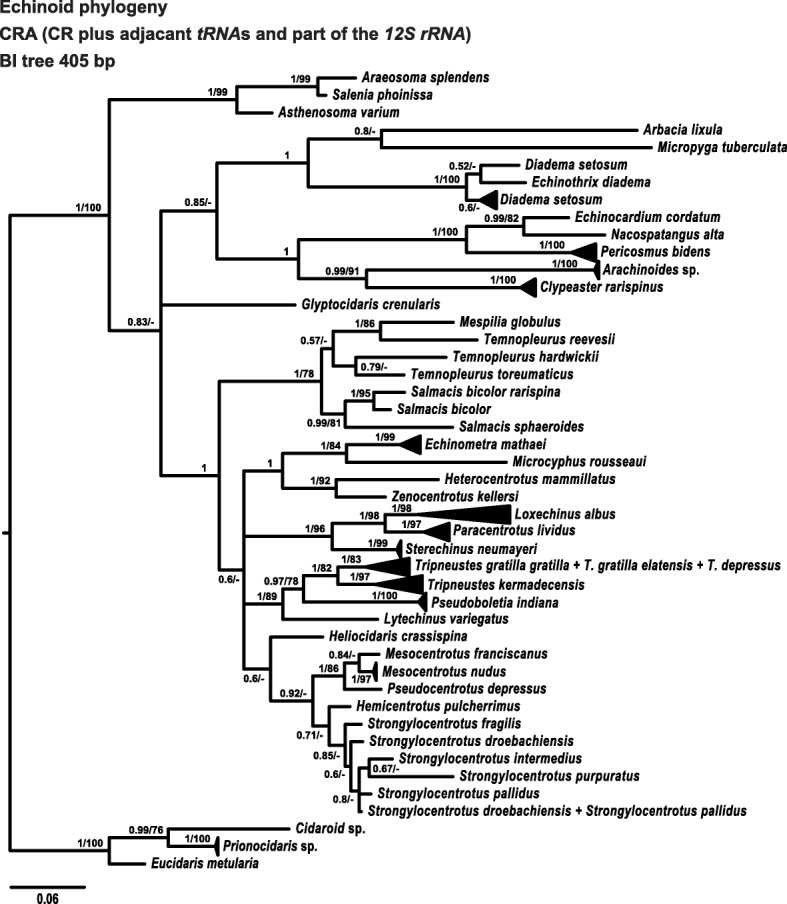
Phylogenetic tree reconstruction of the echinoid control region and adjacent areas (CRA). The BI tree presented is based on 86 unique haplotypes retrieved from a total of 110 sequences, 405 bp long (see Table 1 for details on the sequences used for this tree). Supporting values (> 0.5 posterior probabilities and > 50% ML bootstrap values) are shown above the nodes

Both BI and ML analyses for the complete set of CRA sequences (data set *CRA-all*) rooted on the Cidaridae produced highly congruent topologies for all major clades and subclades. Consequently, BI inferred topologies are presented with both posterior probabilities and bootstrap support (from ML analyses) for each clade (Fig. 4). The strict consensus tree shows good resolution and branch support in most parts of the tree. Most clades received high nodal support except for some members of the Strongylocentroidae and members of the genus *Temnopleurus*. The phylogeny based on the new CRA marker successfully retrieved most putative species as highly supported monophyletic clades. Nevertheless, in several instances the retrieved topology contradicted conventional systematics. The genus *Temnopleurus* for instance, was not monophyletic, as *Temnopleurus reevesii* was retrieved as the sister group of *Mespilia globulus*. Although receiving very poor support for this split, the latter clade formed the sister clade of the other temnopleurids in the current data set, *T. hardwickii* and *T. toreumaticus*. Similarly, sequences of *Diadema setosum* mostly collapsed into a single, well supported clade, although a single *D. setosum* sequence formed the sister of *Echinothrix diadema*. As in the case of *Temnopleurus*, this latter split received very poor nodal support. *Zenocentrotus kellersi* A.H. Clark, 1931 (USNM E40502), is presented here for the first time in a molecular phylogenetic context (Fig. 4). It was recovered as the sister group of *Heterocentrotus mammillatus*, forming with the latter the sister clade of *Echinometra* and *Microcyphus*, in congruence to the current morphological classification.

On two occasions, putative species and subspecies had identical sequences. *Tripneustes gratilla gratilla*, *Tripneustes gratilla elatensis* and *Tripneustes depressus* all shared a single haplotype that was retrieved as sister of *Tripneustes kermadecensis* (Fig. 4). This result was expected, due to the phenomenon of mitochondrial capture between some members of *Tripneustes* [71]. *Strongylocentrotus droebachiensis* and *Strongylocentrotus pallidus* similarly shared a single haplotype, closely related to another sequence of *S. pallidus*. One taxon, *Heliocidaris crassispina*, an echinometrid, was placed as sister group to the family Strongylocentrotidae (in agreement with the *COI* and *16S* trees, but in contrast to current classification), albeit this grouping received very poor nodal support. The only taxon to display markedly contradictory placement (in comparison to current classification) with strong nodal support is a sequence of *Salenia phoinissa* (1/99) that was placed as sister group of *Araeosoma splendens*, in the midst of the expected echinothuriid clade.

## Discussion

**Fig. 5:**
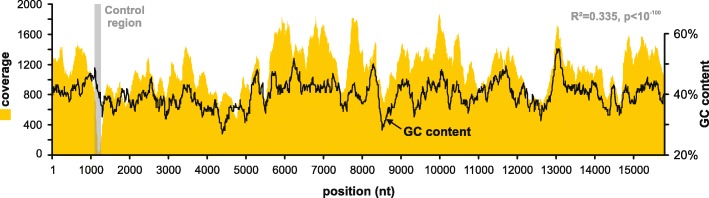
Coverage (orange curve) and GC content (black curve; 200 bp sliding window, 10 bp step width) through the mitogenome of *Hemicentrotus pulcherrimus* (GenBank accession no. KC898202) illustrating moderate (R^2^ = 0.335), but highly significant correlation (t-test, *p* < 10^− 100^) between the two graphs. Note extreme drop of coverage towards the end of the CR (highlighted in grey), which coincides with a slight decrease in GC-content, but shows a much stronger negative excursion than other GC-poor areas in the mitogenome of this species (e.g. at nucleotide positions 4.4, 8.5, or 12.6 kb)

Apart from their use in echinoid phylogeny, the primers used herein can be used as a  complementary tool to NGS assemblies of echinoid mitogenomes, many of which show very low read coverage in the CR (Fig. 1). Coverage in NGS studies was shown to be significantly correlated to GC content of the analysed sequences (e.g., [72] for mitochondrial DNA). The extreme drop in coverage we observed at the echinoid CR, however, is not easily explained as being a direct result of low GC content in this region. Figure 5 illustrates variation in GC contents throughout the mitogenome of *Hemicentrotus pulcherrimus* in relation to coverage. Although reduced coverage generally does seem to coincide with lower GC content, the extreme drop in coverage at the CR could not be explicitly accounted for by the former, as other parts of this echinoid mitogenome show even lower GC content but do not suffer from coverage reduction as substantial as the CR. Similarly, low coverage values have been reported (or can be inferred by read mapping of published data) for the CR of other organisms (insects: [73] [based on mapping of reads from GenBank short read archive SRR835993 on sequence KP216766]; [74]: Fig. 1; crustaceans: [75]: Fig. 1; humans: [76, 77]: 130, Fig. 1). The reasons for this phenomenon are not clear and may be in part related to technical issues (assembly, read mapping) or biochemial qualities of this region (see [3]). Regardless of the driving mechanism, supplementing NGS data with Sanger generated sequences of this target region will improve the quality of mitogenomes assemblies.

By far the most thoroughly studied control region of all organisms is that of humans (see [78]). Although nearly nine times shorter than the human CR and bearing little sequence similarity, several analogies between human and echinoid CR have been proposed [69, 79]. In particular, sequence motifs in the echinoid CR such as the polypurine and polypyrimidine tracts (see above), were regarded as homologous to the mammalian conserved sequence blocks [80, 81]. In line with the interpretations of Jacobs et al. [69], Cantatore et al. [80], and De Giorgi et al. [82], based on merely three echinoid species (*Strongylocentrotus purpuratus*, *Paracentrotus lividus*, and *Arbacia lixula*, respectively), our data from 42 additional echinoid species, tightly conforms to previous observations.

The two most widely used mitochondrial markers, *COI* and *16S*, showed evidence of substitution saturation in our dataset, which may compromise their potential for phylogenetic inference. While the high rate of substitution makes the underlying genes powerful genetic markers, the fast rate of evolution in third codon positions of protein-coding genes, often makes them vulnerable to substantial substitution saturation between highly diverged taxonomic groups [83]. Although we observed substantial saturation at the third codon position in *COI* (Additional file 1: Figure S3 and Table S1), the underlying topologies independently constructed based on codon positions 1 and 2 and codon position 3, respectively, were largely congruent with each other and with tree topology based on morphology. The combination of high topological resolution and lack of substitution saturation of CRA, in contrast, highlights its advantages as a powerful and reliable phylogenetic marker.

Placement of Irregularia in the CRA tree as a sister-group to diadematoids is at odds with the results of recent phylogenetic studies based both on morphology [53, 70] and molecular data [53]. It conforms, however, with earlier phylogenetic hypotheses [84, 85] that resolved irregular echinoids as sister-group to pedinoids and diadematoids. It is worth noting, however, that taxon sampling for irregular echinoids in the present dataset is rather low and more data is needed to verify this result, particularly for basal Irregularia (holectypoids) and holasteroids.

## Conclusions

This study represents the first thorough investigation of the mitochondrial control region across a wide range of echinoid taxa. It provides a tool for complementing incomplete mitochondrial genomes based on NGS experiments. In comparison to the conventional mitochondrial markers such as *16S* and *COI*, the mitochondrial control region of echinoids was found to outperform the traditional markers. As such, it is a powerful, novel marker for phylogenetic inference in echinoids showing high variability, lack of selection, and high compatibility across the entire class.

NGS technologies are revolutionizing our understanding of evolutionary biology, allowing us to generate phylogenetic datasets on the scale of genomes. Nevertheless, Sanger sequencing still offers a rapid, low cost, and accessible sequencing technology that secures its current status as the primary ‘work horse’ of molecular genetics. The present study provides a cheap and efficient tool for species identification in echinoids and for tracking the evolutionary history of the entire class Echinoidea when NGS experiments are beyond the scope of a project.

## Additional file

Additional file 1: **Figure S1.** Gel image showing PCR products. **Figure S2.** Sequencing results shown as a four-color chromatogram. **Figure S3.** Substitution saturation plots. **Table S1.** Substitution saturation analysis. (PDF 1735 kb)

## Abbreviations

5’–UTR: Five prime untranslated region
AIM: Auckland War Memorial Museum
BI: Bayesian Inference
BIC: Bayesian Information Criterion
bp: Base pairs
CAS: California Academy of Sciences
COI: Cytochrome c oxidase subunit I
CpG: 5’–C–phosphate–G–3’
CR: Control region
CRA: Control region and adjacent tRNAs
ML: Maximum Likelihood
MNHN: Muséum national d'Histoire naturelle
mtDNA: Mitochondrial DNA
NGS: Next generation sequencing
NHMW: Natural History Museum Vienna
PP: Posterior probabilities
QM: Queensland Museum
rRNA: Ribosomal RNA
SMNH: Steinhardt Museum of Natural History
SNP: Single nucleotide polymorphism
tRNA: Transfer RNA
USNM: Smithsonian Institution National Museum of Natural History
