# Supplementary material for: Mind the gap! The mitochondrial control region and its power as a phylogenetic marker in echinoids
Source: BMC Evol Biol. 2018 May 30;18:80. doi: 10.1186/s12862-018-1198-x (PMC5977486; doi:10.1186/s12862-018-1198-x)
Supplement: Supplementary file 1 — Figure S1. Gel image showing PCR products. Figure S2. Sequencing results shown as a four-color chromatogram. Figure S3. Substitution saturation plots. Table S1. Substitution saturation analysis. (PDF 1735 kb) [file 12862_2018_1198_MOESM1_ESM.pdf]

Mind the Gap! The mitochondrial control region and its power as a phylogenetic marker in echinoids

Authors

Omri Bronstein*[,a,b]

Andreas Kroh[a]

Elisabeth Haring[b,c]

Affiliation

[a] Natural History Museum Vienna, Geological-Paleontological Department, 1010 Vienna, Austria

[b] Natural History Museum Vienna, Central Research Laboratories, 1010 Vienna, Austria

[c] University of Vienna, Department of Integrative Zoology, Vienna, Austria

* Corresponding author

E-mail address: omribronstein@gmail.com

Tel: +43-1-52177-610

Fax: +43-1-52177-459

Keywords:

Control region; Molecular phylogeny; Mitochondrial markers; NGS; Echinoidea

Fig. S1 Inverted tone image of an ethidium-bromide-stained agarose gel (1.5%), showing PCR products from the amplification (using the CRA primer pair: CR15fwd and CR08rev as discussed in the text) of various echinoid species. L denotes DNA ladder. Lane 1 – *Tripneustes gratilla elatensis*; 2 – *Diadema setosum*; 3 – *Lytechinus variegatus*; 4 – *Tripneustes kermadecensis*; 5 – *Clypeaster rarispinus*; 6 – *Echinometra* sp.; 7 – *Periocosmus bidens*; 8 – *Eucidaris metularia*; 9 – *Paracentrotus lividus*; 10 – *Araeosoma splendens*; 11 – *Salmacis bicolor*; 12 – *Prionocidaris* sp.

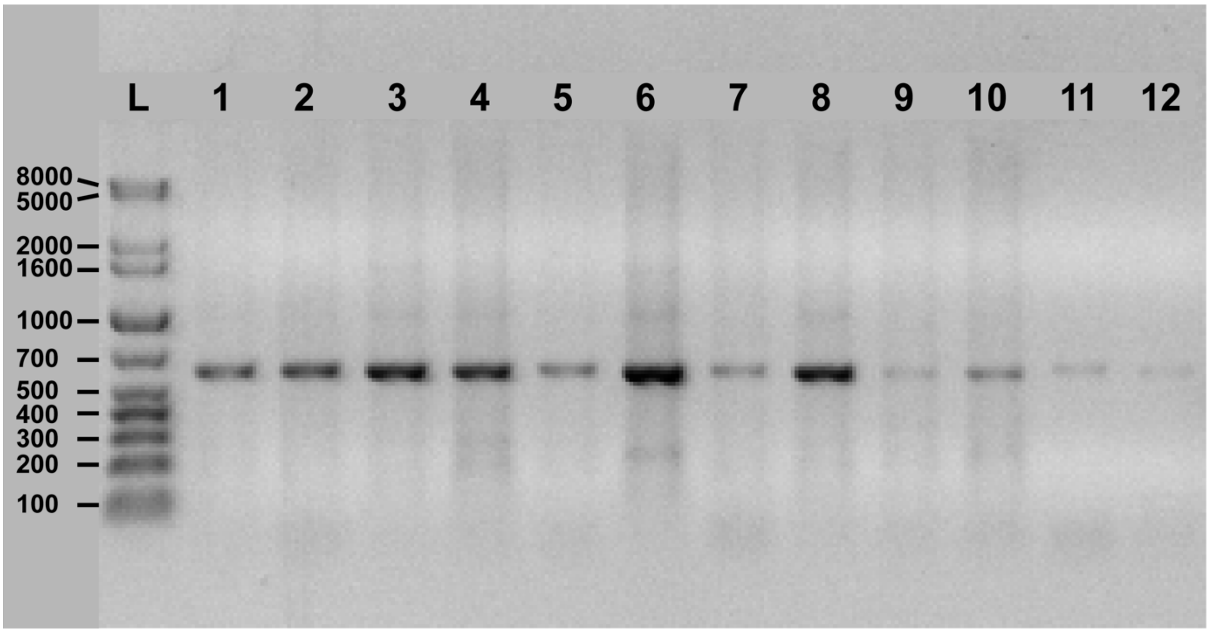

Fig. S2 Sequencing results shown as a four-color chromatogram of a portion of the CR adjacent to the guanine stretch (Poly-G stretch) from a specimen of *Tripneustes gratilla*. Sequencing signal drops abruptly and sequencing does not continue past this region.

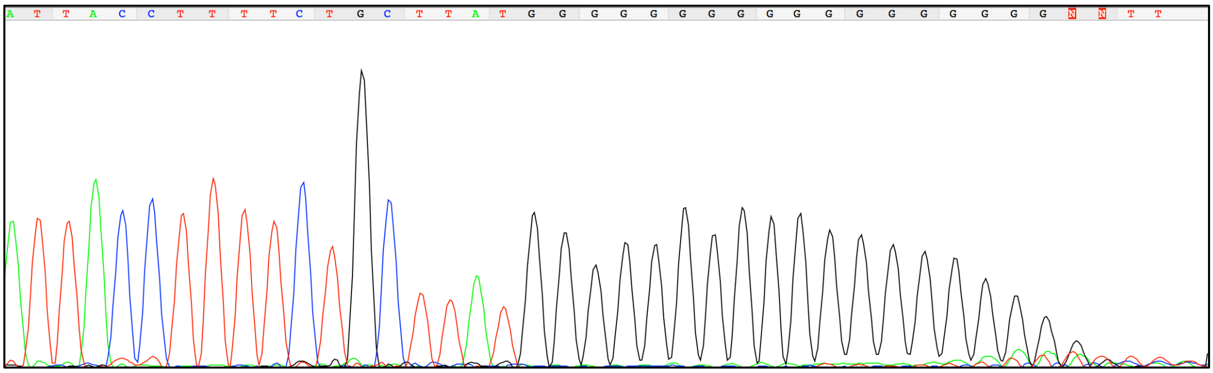

Fig. S3 Substitution saturation plots. The number of transitions (s) and transversions (v) is plotted against F84 genetic distance (Felsenstein, 1984). **A** – *COI* codon position 1&2; **B** – *COI* codon position 3; **C** – 16S; **D** – CRA.

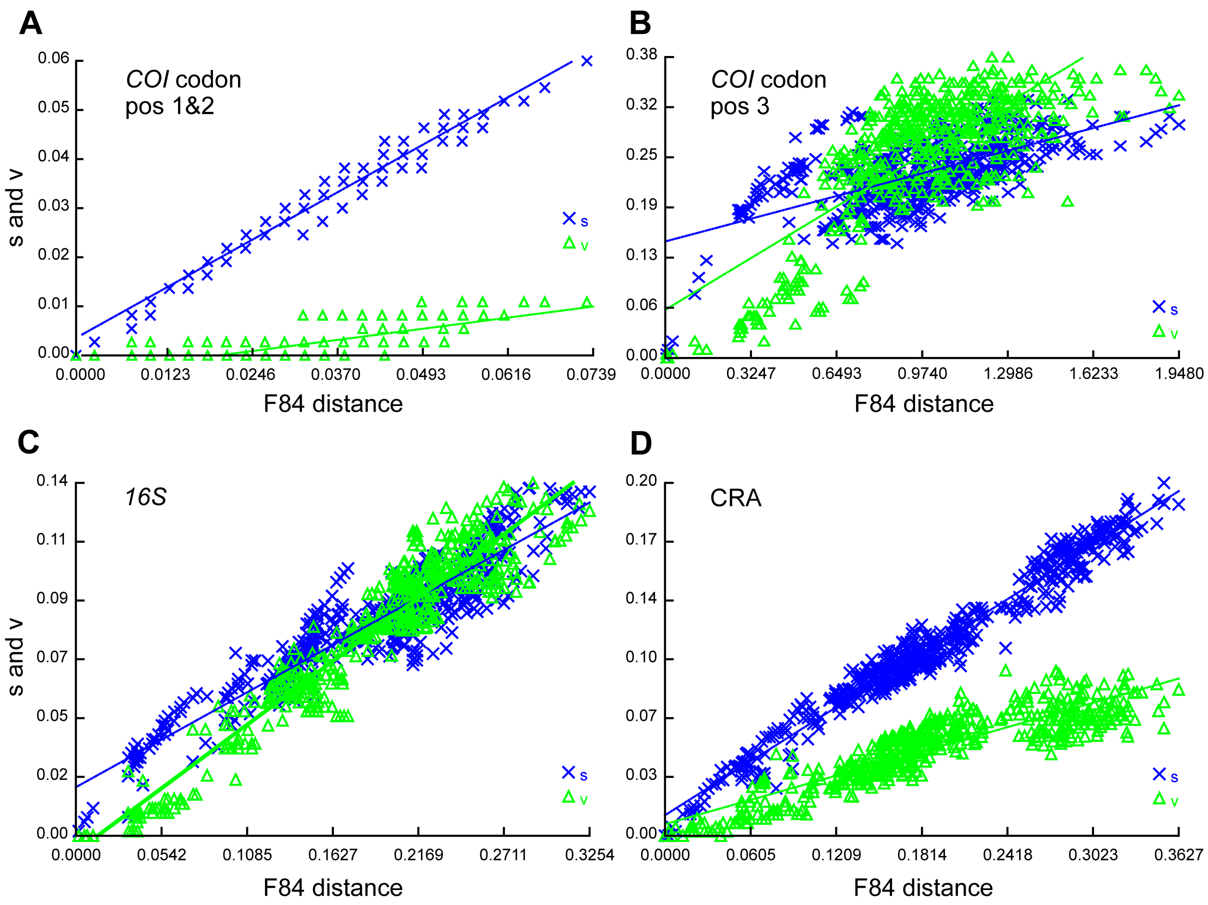

Table S1 Substitution saturation analysis for the different markers based on the index of substitution saturation (Xia et al., 2003; Xia and Lemey, 2009), as implemented in DAMBE6 (Xia, 2017).

Marker dataset	Number of OTU[a]	Iss[b]	$I_{ss.cSym}$[c]	df[d]	*p* value[e]	$I_{ss.cAsym}$[f]	df[d]	*p* value[e]
Mito-COI Codons 1&2								
	4	0.054	0.786	244	<0.0001	0.756	244	<0.0001
	8	0.055	0.740	244	<0.0001	0.629	244	<0.0001
	16	0.054	0.697	244	<0.0001	0.488	244	<0.0001
	32	0.053	0.688	244	<0.0001	0.359	244	<0.0001
Mito-COI Codon 3								
	4	0.665	0.777	177	0.0032	0.772	177	0.0050
	8	0.685	0.737	177	0.1348	0.642	177	0.2086
	16	0.681	0.637	177	0.1607	0.454	177	<0.0001
	32	0.684	0.695	177	0.7165	0.386	177	<0.0001
Mito-16S								
	4	0.327	0.799	351	<0.0001	0.767	351	<0.0001
	8	0.356	0.757	351	<0.0001	0.646	351	<0.0001
	16	0.359	0.731	351	<0.0001	0.520	351	<0.0001
	32	0.363	0.708	351	<0.0001	0.380	351	0.5882
Mito-CRA								
	4	0.248	0.789	333	<0.0001	0.757	333	<0.0001
	8	0.267	0.743	333	<0.0001	0.631	333	<0.0001
	16	0.273	0.703	333	<0.0001	0.494	333	<0.0001
	32	0.274	0.692	333	<0.0001	0.363	333	0.0021

[a] Number of sequences used in the random resampling.
[b] index of substitution saturation.
[c] critical value for a symmetrical tree topology.
[d] degrees of freedom.
[e] probability that I_{ss} is significantly different from the critical value ($I_{ss.cSym}$/$I_{ss.cAsym}$).
[f] critical value for an asymmetrical tree topology.

Note: two-tailed tests are used.
